# Crawford Williamson Long and Ether

**Published:** 1917-12

**Authors:** E. M. Magruder

**Affiliations:** Charlottesville, Va.


### Journal-Record of Medicine

Successor to Atlanta Medical and Surgical Journal, Established 1855
and Southern Medical Record, Established 1870

**OWNED BY THE ATLANTA MEDICAL JOURNAL COMPANY**

PUBLISHED MONTHLY

*Official Organ Fulton County Medical Society, State Examing Board, Atlanta, Birmingham and Atlantic Railroad Surgeon's Association, Chattahoochee Valley Medical and Surgical Association, Etc.*

A. W. STIRLING, M. D., C. M., D. P. H.; J. S. HURT, B. Ph., M.D.
GEO. M. NILES, M. D., W. J. LOVE, M. D., (Ala.); Associate Editors

E. W. ALLEN, Business Manager

**COLLABORATORS**

W. F. WESTMORELAND, M. D., General Surgery
F. W. McRAE, M. D., Abdominal Surgery
ALLEN H. BUNCE, Medical Societies
E. B. BLOCK, M. D., Diseases of the Nervous System
MICHAEL HOKE, M. D., Orthopedic Surgery
CYRUS W. STRICKLER, M. D., Legal Medicine and Medical Legislation
E. C. DAVIS, A. B., M. D., Obstetrics
E. G. JONES, A. B., M. D., Gynecology
ALLEN H. BUNCE, M. D., Pathology and Bacteriology
R. T. DORSEY, Jr., B. S., M. D., Medicine
L. M. GAINES, A. B., M. D., Internal Medicine
GEO. C. MIZELL, M. D., Diseases of the Stomach and Intestines
L. B. CLARKE, M. D., Pediatrics
EDGAR PAULLIN, M. D., Opsonic Medicine
THEODORE TOEPEL, M. D., Mechano Therapy
E. G. BALLENGER, M. D., Diseases of the Genito-Urinary Organs

VOL. LXIV. ATLANTA, GA., DEC., 1917. NO. 9

### CRAWFORD WILLIAMSON LONG AND ETHER.*

**By E. M. Magruder, M.D., Charlottesville, Va.**

(Continued)

The claims of Wilhite, Marcy, and Sir James Y. Simpson, which were also once put forward, are so ill founded, flimsy, and ridiculous, that they were never seriously considered and

*Paper read before Interstate Association of Anesthetists, at Louisville, Ky., July 26, 1916.—Reprinted from International Journal of Surgery.

may be dismissed with mere mention. Simpson, however, was the first to discover the anesthetic powers of chloroform and the first to use it in obstetrics; but this occurred in 1847, five years after Long's discovery.

In 1846 and 1849 Morton applied to Congress for an appropriation as a reward for the discovery of the anesthetic effects of sulphuric ether, and Jackson and the representatives of Wells, who had died in 1848, combatted this claim; this gave rise in Congress to the "Ether Controversy," that raged till 1854, and in which Long took no part. In the latter year, however, persuaded by his friends, Long wrote a letter to United States Senator Dawson, of Gerogia, giving an account of his work and claiming priority in the discovery and use of surgical anesthesia.

At the request of Dawson, Jackson then visited Long in Athens, March 8, 1854, investigated his claims, and was so impressed by them that he proposed that they share the honor and benefits of the discovery by uniting their claim against those of Wells and Morton—Jackson to claim the discovery and Long the first practical use of surgical anesthesia; but this was declined by Long, who claimed that he was the first both to discover and use surgical anesthesia, and Jackson then acqnowledged the justice of Long's claims, using these words: "Well, Doctor, you have the advantage of us other claimants in the first discovery and use of sulphuric ether as an anesthetic; but we have the advantage of having first published it to the world." (See letter of Capt. C. H. Andrews, who witnessed the interview between Long and Jackson.)

Jackson also immediately wrote to Senator Dawson acknowledging that Long had undoubtedly used ether in surgery before any of the claimants for the appropriation and withdrew from the contest.

On April 11, 1861, Jackson published a paper in the Boston Medical and Surgical Journal in which he gave Long the credit for prior discovery and use, employing this language: " Had he (Long) written to me in season I would have presented his claims to the Academy of Sciences of France; but he allowed his case to go by default and the Academy knew no more of his claims than I did.''

The interviews between Long and Jackson were perfectly pleasant and amicable and long was most fair in his dealing with the latter, as is shown by the following: When preparing, in 1854, to publish a paper containing the proofs of his claims, he wrote to his rival, Jackson, in this style: ''Ours are rival claims, and permit me, Sir, to say that, although our claims are conflicting, I would not knowingly say anything in the article which would be displeasing to you. I entertain high respect for you as a gentleman and man of science and feel honored by your acquaintance. Still it becomes each one of us to use all honorable means to advance his own claims. Shall it meet with your approval I may refer to your admission to Hon. W. C. Dawson and myself of the belief of the correctness of my claims; I will, however, make no allusion to your letter to Mr. Dawson or to the conversation held with myself unless it meets with your sanction.''

On April 19, 1854, an appropration bill entitled ''An Act to recompense the discoverer of anesthesia,'' containing the names of Jackson, Wells, Morton, and Long, was passed by the United States Senate. In order to determine which of these persons was the original and true discoverer, the bill further directed proceedings to be instituted in the United States Circuit Court of Northern New York, but this was never done.

On April 21, the same bill was before the House, but was ''laid on the table,'' awaiting further developments. But Long failed to press his claims and no appropriation was ever made.

His appearance in the Congressional arena was simply to defeat the plans of his rivals which, if successful, would have tended to deprive him of his rights. Having accomplished this he abstained from urging his claims further before Congress, as he did not consider that the proper body to decide the question of the discovery nor did he wish pecuniary reward, his own desire being recognition by the medical profession. His own words vividly express his views as follows:

"My only wish about the discovery of anesthesia is to be known as a benefactor of my race. I am free to say that I would much prefer my claim to the discovery being admitted by the National (American) Medical Association to any appropriation by Congress." (See letter to Dr. P. F. Eve, and also Long's first paper and affidavits of Rawls, Dr. Groves, James M. Venable, Dr. Carton, Dr. Camak, etc.)

He wished to lay his claims before the American Medical Association, but was informed that no controversy could be settled by that body. The latter, however, passed a resolution of scathing censure against Morton on account of his patent (Transactions of American Medical Association, Vol. XV., p. 53, also "Medicine in America"), and the Journal of the American Medical Association for October 30, 1915, contains an article in which the credit for the great discovery is given to Long and all the false accusations against him are duly exposed.

It seems proper in passing to notice the arguments used by Long's opponents to discredit his claims.

The chief argument was that: "Long made no publication of his experiments until December, 1849, three years after the universal publication and adoption of surgical anesthesia."

The truth of this depends upon the meaning of the word "Publication." Webster says "Publication" means, "Notifications of the people at large, either by words, writing, or printing."

While it is true that Long did not write and publish his work in any periodical until December, 1849, it is nevertheless also true, that he did from the first publish his discovery verbally and in other ways which are legitimate, though not so effective:

(1) By always operating in the presence of witnesses.

(2) By making no effort to keep secret either the agent inhaled or the results.

(3) By encouraging publicity by public discussion and by urging the employment of ether by other pysicians, so that it was known over a wide section of country.

Neither Wells, Jackson, nor Morton, ever published anything upon the subject of their discoveries, this being done by Bigelow, while Wells not only failed in his two attempts at public demonstration of his, but finally abandoned it; and Jackson and Morton used their utmost endeavor by means of a patent to keep from the public and profession the nature of the agent inhaled.

Thus it will be seen that while Long freely and voluntarily gave his discovery to the world, Morton and Jackson endeavored to keep their secret, and not only never published anything upon the subject but were forced to disclose the nature of the agent inhaled. Likewise, Morton, living in the large and cultured city of Boston, with unrivaled journalistic facilities, the use of a fine hospital, and eminent surgeons to aid him, was admirably prepared for universal publication; while Long, whose home was in a small village with none of the above facilities and with a practice in which surgery was exceedingly rare, was but poorly equipped for publication. The nearest hospital, medical journal, and railroad were at Augusta, Georgia, 140 miles distant, and the nearest newspaper, a weekly, was at Athens, 20 miles away.

But while Long had no hospital in which to exploit his discovery of anesthesia and no coterie of eminent surgeons to applaud, encourage, and publish his work as Morton had, his operations were as public as his meager facilities would permit, being performed either in his office or in private homes, and always in the presence of witnesses, though his cramped quarters would admit but few at a time. (Affidavits of the Venables, Rawls, the Thurmonds, the Hayes, the Vinsons, Thompson, Bailey, Ware, Dr. De LaPerriere, Dr. Carlton, Dr. Camak, Dr. Groves, Mrs. Carlton, Liddell, Collins, Pendergrass, Henderson, and dozens of others.)

The following incidents show that Long's discovery was known to the public and the medical profession at large of Georgia:

The famous writer, Charles Smith, "Bill Arp," states that in 1842, while attending Franklin College at Athens, he heard Dr. Long's discovery of ether anesthesia discussed by the professor of chemistry publicly before his class.

In May, 1843, Dr. R. D. Moore, of Athens, amputated a leg and said to his three student assistants: "If I had thought of it before leaving home I would have tried Dr. Crawford W. Long's great discovery, producing insensibility by the inhalation of ether." This operation was performed with the patient under the influence of morphin. (Affidavits of Drs. Carlton and Camak, who were Moore's assistants.)

In November or December, 1844, Dr. J. B. Carlton, of Athens, visited Long at Jefferson for the purpose of discussing with him his discovery and finding out more about it. While there he was induced by Long to administer ether to a patient in his office and extract a tooth (affidavit of Mrs. Carlton). This was the only instance in which ether was used by any other physician in that section, as those who heard of it feared fatal effects; none of the Athens physicians employed it.

The other arguments used against Long were: That he did not himself administer the ether but had it administered by the patient; that the anesthesia was not carried beyond the stage of exhilaration; that he did not carry his experiments far enough to reach a decided result; that he did not seem to appreciate the great value of his discovery and abandoned it; and that he presented his claims before Congress but his evidence failed to convince the House.

The most superficial examination of Long's records, now in my hands, is sufficient to prove beyond the least doubt that these accusations had absolutely no foundation in fact and originated in desperation at Long's inevitable triumph before the world. Did space permit I could bring forward overwhelming evidence, but it will suffice to say it can easily be proved that Long always administered the ether himself to complete anesthesia; that he did reach most decided results as he operated without pain, and showed the highest appreciation of the value of the discovery by continuing to employ it in minor and major operations, and even in obstetrics, to the time of his death; and that his appearance before Congress was only to checkmate his rivals and not to gain recognition or pecuniary rewards.*

Long's first operation with ether (March 30, 1842) was about two and a half years before Well's discovery (December 11, 1844), and about four and a half years before Morton's (September 30, and October 16, 1846). He likewise performed at least six minor operations under ether anesthesia before Wells made his experiments, and at least seven before Morton's operation. He afterwards used ether in major operation, as amputations of limbs and removal of the female breast for cancer; in the latter he scraped the ribs and removed the axillary glands (the modern Halstead's operation), and never had a cancer to return; he refused to operate, however, after the disease had reached the skin. One of these patients lived sixty years after the operation, with no return of the trouble.

In his operations he employed carbolic acid and tincture of iodin as antiseptics, being thus far ahead of the time.

For the first operation on Venable, Long, having previously promised to do the work for a nominal fee, charged two dollars including the cost of the ether, which was 25 cents. "He never charged over one hundred dollars for a breast operation." (Da Costa.)

Long undoubtedly made a great mistake in not at once writing up and publisihng, in some medical journal, the results of his experiments; but it was December, 1849, nearly eight years after his first operation with ether, before he did this, and in the meantime, Wells and Morton made their discovery, while Bigelow published Morton's experiments and the surgical work in the Massachusetts General Hospital resulting therefrom.

His procrastination for many years caused difficulty in established his claims, as the discovery came to the knowledge of the world at large through the publication by Bigelow of Morton's work, and in this way Morton's name was at first connected with the discovery instead of Long's.

The causes of Long's delay in publishing his discovery, as explained by himself, are as follows: :

I. "I was anxious, before making my publication, to try etherization in a sufficient number of cases to fully satisfy my mind that anesthesia was produced by the ether and was not the effect of the imagination or due to any idiosyncrasy, etc. Those high in authority were advocating the mesmeric state as adequate to prevent pain in surgical operations. Notwithstanding thus sanctioned I was an unbeliever in the science and of the opinion that, if the mesmeric state could be produced at all, it was only in those of strong imagination and weak minds, and was to be ascribed solely to the imagination."

II. "After fully satisfying myself of the power of ether to produce anesthesia, I was desirous of administering it in a severer surgical operation than any I had performed. In my practice prior to the published account (by Bigelow) of the use of ether as an anesthetic I had no opportunity of experimenting with it in a capital operation. Surgical operations are not of frequent occurrence in a country practise and especially in the practice of a young physician.

"While I was cautiously experimenting with ether as cases occurred with the view of fully testing its anesthetic effects and its applicability to severe as well as minor surgical operations, others more favorably situated engaged in similar experiments and consequently the publication of etherization did not bide my time."

III. Opposition from the older physicians of the vicinity and the ignorance and superstition of the public prevented his using ether as often as he might have done. It was frequently said " he would kill some one with it yet." "The sleep had too much the semblance of the sleep that knows no waking."

He received no encouragement to persist in the use of ether from any one except his young wife, whom he married at the age of sixteen, the year of his discovery.

With regard to the discovery Long was exceedingly modest, as is shown by the following:

"I assume no particular credit to myself for making the discovery at the time, and I am astonished that is was not made earlier. Witnessing the facts that presented themselves, I was almost forced by the facts observed to apply my knowledge to the use of the article in surgical operations."

There was no universal recognition of the work and claims of Dr. Long till 1877, when Dr. J. Marion Sims of New York, himself a South Carolinian, had his attention called to the matter by Dr. Phillip A. Wilhite of South Carolina, who had been a student of Dr. Long and was then in New York attending a private operation of Dr. Sims. Sims became convinced of the merit of Long's claims and published a paper in the Virginia Medical Monthly of May, 1877, demanding their recognition. Soon after this Long began to receive letters from physicians and surgeons all over the world, and his declining years were gladdened by world-wide recognition of his claims to an achievement most dear to his heart and one which stands out supreme over all.

Time has proved a just and righteous judge, the things of Caesar have been rendered unto Caesar, virtue has had its reward, and Crawford W. Long will go down in history as one of the "Seven Wise Men of Medicine," one of the seven epoch-makers of medicine, along with Harvey (the circulation), Jenner (Vaccination), Prevez (Hypodermic Medication), Pasteur (the Germ Theory), Lister (Antiseptic Surgery), Von Behring (Serum Therapy)—The seven greatest benefactors of the human race, and Long the greatest of the seven.

